# An Overview to Molecularly Imprinted Electrochemical Sensors for the Detection of Bisphenol A

**DOI:** 10.3390/s23208656

**Published:** 2023-10-23

**Authors:** Ying Pan, Mengfan Wu, Mingjiao Shi, Peizheng Shi, Ningbin Zhao, Yangguang Zhu, Hassan Karimi-Maleh, Chen Ye, Cheng-Te Lin, Li Fu

**Affiliations:** 1 College of Materials and Environmental Engineering, Hangzhou Dianzi University, Hangzhou 310018, China; panying@hdu.edu.cn; 2 Qianwan Institute, Ningbo Institute of Materials Technology and Engineering (NIMTE), Chinese Academy of Sciences, Ningbo 315201, China; wumengfan@nimte.ac.cn (M.W.); shimingjiao@nimte.ac.cn (M.S.); shipeizheng@nimte.ac.cn (P.S.); zhaoningbin@nimte.ac.cn (N.Z.); zhuyangguang@nimte.ac.cn (Y.Z.); 3 Key Laboratory of Marine Materials and Related Technologies, Zhejiang Key Laboratory of Marine Materials and Protective Technologies, Ningbo Institute of Materials Technology and Engineering (NIMTE), Chinese Academy of Sciences, Ningbo 315201, China; 4 University of Chinese Academy of Sciences, 19 A Yuquan Rd., Shijingshan District, Beijing 100049, China; 5 School of Resources and Environment, University of Electronic Science and Technology of China, Chengdu 611731, China; hassan@uestc.edu.cn; 6 School of Engineering, Lebanese American University, Byblos 1102-2801, Lebanon

**Keywords:** molecularly imprinted polymers, electrochemical sensors, bisphenol A, selectivity, sensitivity, on-site detection

## Abstract

Bisphenol A (BPA) is an industrial chemical used extensively in plastics and resins. However, its endocrine-disrupting properties pose risks to human health and the environment. Thus, accurate and rapid detection of BPA is crucial for exposure monitoring and risk mitigation. Molecularly imprinted electrochemical sensors (MIES) have emerged as a promising tool for BPA detection due to their high selectivity, sensitivity, affordability, and portability. This review provides a comprehensive overview of recent advances in MIES for BPA detection. We discuss the operating principles, fabrication strategies, materials, and methods used in MIES. Key findings show that MIES demonstrate detection limits comparable or superior to conventional methods like HPLC and GC-MS. Selectivity studies reveal excellent discrimination between BPA and structural analogs. Recent innovations in nanomaterials, novel monomers, and fabrication techniques have enhanced sensitivity, selectivity, and stability. However, limitations exist in reproducibility, selectivity, and stability. While challenges remain, MIES provide a low-cost portable detection method suitable for on-site BPA monitoring in diverse sectors. Further optimization of sensor fabrication and characterization will enable the immense potential of MIES for field-based BPA detection.

## 1. Introduction

BPA is a synthetic organic compound that has become a topic of global concern in recent years. The reason for this widespread attention is its extensive use in various industries and the potential harmful effects it can cause to both human health and the environment [1,2]. BPA is an organic compound characterized by two phenol functional groups attached to a central carbon atom. This structure has earned it the term ‘bisphenol’. BPA is primarily used in the production of polycarbonate plastics and epoxy resins [3,4,5]. These materials are common in an array of consumer products, ranging from food and beverage containers, water bottles, and thermal paper to medical devices and compact discs [6]. The widespread use of BPA has made it an integral part of modern life, virtually surrounding us in our daily routines [7].

The physical properties of BPA, such as its poor solubility in water but good solubility in organic solvents, make it a versatile component in various manufacturing processes [8]. Its stability and resistance to immediate degradation contribute to its persistence in the environment, making its monitoring and detection critically important [9]. The prevalence of BPA in everyday products has led to widespread human exposure. The primary route of exposure is dietary ingestion, although dermal contact and inhalation are also possible [10]. BPA is considered an endocrine disruptor, capable of mimicking estrogen and interfering with hormonal functions. This interference can lead to a plethora of potential health effects including reproductive disorders, cardiovascular diseases, metabolic disorders such as diabetes, and neurodevelopmental issues in children [11]. From an environmental perspective, BPA poses significant risks. It can leach into soil and water systems from discarded products or industrial waste, causing harm to aquatic life and disrupting ecosystems [12]. The environmental persistence of BPA, combined with its potential bioaccumulation and biomagnification in wildlife, underlines the urgency of its detection and monitoring.

The potential risks associated with BPA exposure underscore the importance of its detection. Accurate, reliable, and rapid detection methods are essential not just for ensuring regulatory compliance, but also for preventing excessive human exposure and mitigating environmental impact [13]. Monitoring BPA levels in various matrices can provide valuable data for epidemiological studies investigating the health effects of BPA exposure, thereby informing risk assessments and public health interventions [14]. Numerous methods have been developed over the years for BPA detection, including high-performance liquid chromatography (HPLC) [15], gas chromatography–mass spectrometry (GC-MS) [16], immunoassays [17], and fluorescence detection [18]. While these methods are generally reliable and sensitive, they often require sophisticated, expensive equipment and extensive sample preparation. Additionally, they necessitate skilled personnel and time-consuming procedures, which limit their applicability for on-site, real-time detection.

**Figure 1:**
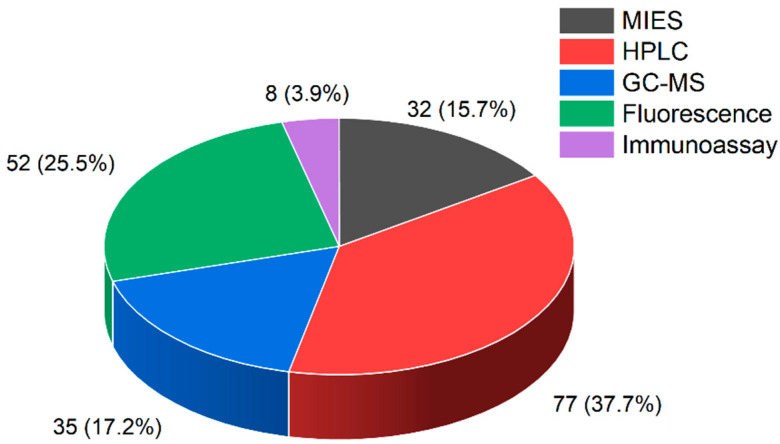
Bar chart of papers published in the last 5 years with regard to BPA detection using either MIES, HPLC, GC-MS, fluorescence method or immunoassay.

In response to these challenges, MIES have emerged as a promising alternative for BPA detection. Molecular imprinting creates selective recognition sites in the polymer matrix that are complementary to the target analyte in size, shape, and chemical functionality [19,20,21,22]. This allows MIES to offer high selectivity towards BPA even in complex sample matrices [23,24,25]. For example, Zhang et al. [26] developed a MIES that showed excellent selectivity for BPA over structurally similar compounds like bisphenol AF, tetrabromo BPA and hydrogenated bisphenol A. The transducing mechanism of MIES also enables rapid, portable and cost-effective detection. The binding of BPA to the imprinted sites induces changes in electrical properties like current, potential or impedance. By measuring these signals, BPA can be reliably detected within minutes using simple instrumentation. MIES have been integrated into portable devices for on-site analysis. For example, Beduk et al. [27] presented a novel portable, wireless electrochemical sensor using laser-scribed graphene (LSG) electrodes to detect BPA. The key innovation is the integration of a homemade portable Bluetooth-connected potentiostat device with the LSG sensor, enabling wireless on-site BPA detection without bulky equipment. Moreover, MIES demonstrate high sensitivity for BPA, with detection limits comparable to or better than conventional methods. Figure 1 shows a bar chart of papers published in the last 5 years with regard to BPA detection using either MIES, HPLC, GC-MS, the fluorescence method or immunoassay. The data were collected from Web of Science core collection databases. Over the past five years, there have been 204 papers published on the detection of BPA. The most common detection method was HPLC, which accounted for 77 papers or about 38% of the total. The second most common method was fluorescence, which accounted for 52 papers or 25% of the total. MIES was also a significant detection method, accounting for 32 papers or 16% of the total. While not the most prevalent technique, MIES still represented a substantial proportion of recent BPA detection research.

This review aims to provide a comprehensive overview of MIES for BPA detection. It delves into the principles of molecular imprinting and electrochemical sensing, the fabrication process, and the performance evaluation of these sensors. The review also highlights recent advances and future perspectives in this field, including innovations in materials and fabrication techniques, integration with other technologies, and potential challenges and opportunities. The goal is to inform researchers, industry professionals, and policymakers about the current state of this technology and its potential for future applications.

## 2. Molecularly Imprinted Electrochemical Sensors

MIES have emerged as a promising tool for the detection of BPA due to their high selectivity, sensitivity, and cost-effectiveness. The principle behind these sensors lies in the unique combination of molecular imprinting and electrochemical detection.

### 2.1. Principle of MIES for BPA Detection

**Figure 2:**
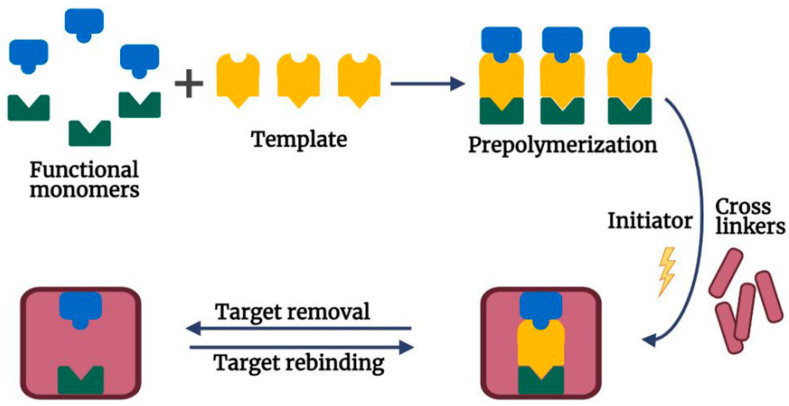
Schematic illustration of the process of molecular imprinting and target removal/rebinding. Reprinted with permission from Elsevier from Ref. [28].

The process of molecular imprinting involves the creation of specific recognition sites within a polymer matrix that can selectively bind to a target molecule, in this case, BPA. This is achieved via the self-assembly of functional monomers around a template molecule, followed by the polymerization process that solidifies the structure. The template molecule is then removed, leaving behind cavities or imprints that are complementary to BPA in terms of shape, size, and functional groups (Figure 2).

The selection of monomers is a critical step in molecular imprinting as it significantly affects the recognition properties of the resulting molecular imprinted polymer (MIP). Monomers are chosen based on their ability to form non-covalent interactions with BPA. For example, methacrylic acid (MAA) is commonly used as a functional monomer due to its ability to form hydrogen bonds with BPA. For example, Wang et al. [29] fabricated an MIP receptor using MMA as the primary functional monomer for imprinting. Compared to conventional MMA-only MIP sensors, the dual-functionality receptor exhibited dramatically improved sensitivity and lower noise when detecting BPA. Another example is 4-vinylpyridine (4-VP), which can interact with BPA via π–π interactions. Ekomo et al. [30] synthesized the MIP using ferrocenylmethyl methacrylate as a redox-active monomer, 4-VP as a co-monomer, and ethylene glycol dimethacrylate (EGDMA) as a crosslinker. Among them, MAA showed the lowest (most negative) interaction energy with BPA based on DFT investigation, indicating the strongest binding [31].The ratio of monomer to template also plays a crucial role in determining the quality of the imprints. An optimal ratio ensures sufficient interaction between the monomer and BPA, leading to effective imprint formation. For instance, a study by Xu et al. [32] demonstrated that a molar ratio of 5:1 (MMA:BPA) resulted in MIPs with high selectivity and affinity for BPA.

Electrochemical detection is a powerful analytical technique that measures the changes in electrical signals upon interaction with a target analyte [33,34,35,36,37,38,39,40,41]. In the context of MIES for BPA detection, the rebinding of BPA to the imprinted cavities alters the electrical properties of the sensor, which can be quantified and correlated to the concentration of BPA.

There are three main types of electrochemical detection methods: potentiometry, amperometry/voltammetry, and electrochemical impedance spectroscopy. Potentiometric sensors measure the potential difference between two electrodes, which changes upon BPA binding. Amperometric/voltammetric sensors monitor the current resulting from a redox reaction involving BPA. For example, Huang et al. [42] reported a MIES for detecting BPA. A key advantage of the sensor is the use of amperometry for detection. This electrochemical technique applies a constant potential and measures the resulting current, which is proportional to BPA concentration. The linear detection range was 8.0 × 10^−6^ to 6.0 × 10^−2^ M, with a low limit of detection of 1.38 × 10^−7^ M. The choice of detection method depends on several factors, including the properties of BPA, the design of the sensor, and the specific application requirements. An amperometric/voltammetric sensor might be preferred for its high sensitivity in detecting low concentrations of BPA, while a potentiometric sensor might be chosen for its simplicity and ease of use.

In summary, molecular imprinting involves creating specific recognition sites in a polymer matrix that can selectively bind to BPA. This is performed via self-assembly of functional monomers around a BPA template molecule. The template is then removed, leaving behind cavities complementary to BPA. An optimal ratio ensures sufficient interaction for effective imprint formation. Electrochemical detection measures changes in electrical signals upon BPA rebinding to imprinted cavities. Potentiometry, amperometry/voltammetry and impedance spectroscopy are commonly used detection methods.

### 2.2. Fabrication Process

The fabrication of MIES for BPA detection involves two main steps: synthesis of MIPs and integration of MIPs with electrochemical sensors. The synthesis of MIPs begins with the formation of a pre-polymerization complex between the BPA and functional monomers. This process is driven by non-covalent interactions such as hydrogen bonding, van der Waals forces, or π–π interactions [43,44]. For example, MAA, a common functional monomer, can form hydrogen bonds with BPA [45], while 4-VP can interact via π–π interactions [46].

The pre-polymerization complex is then polymerized in the presence of a cross-linker and an initiator. The cross-linker provides structural stability to the polymer matrix, while the initiator triggers the polymerization reaction. For instance, ethylene EGDMA is frequently used as a cross-linker due to its excellent cross-linking ability [45,47,48], and azobisisobutyronitrile (AIBN) is a typical initiator used in thermal initiation [48,49,50].The polymerization process can be carried out using various techniques such as bulk polymerization [51], precipitation polymerization [30], or emulsion polymerization [52,53]. The choice of technique depends on the desired properties of the MIPs, such as particle size and shape, porosity, and binding capacity.

**Figure 3:**
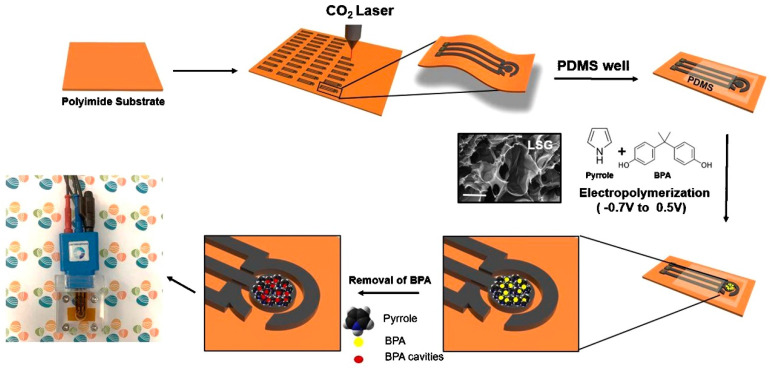
One-step electrosynthesized MIP on LSG for BPA sensing. Reprinted with permission from Elsevier from Ref. [56].

The integration of MIPs with electrochemical sensors is a crucial step in the fabrication of MIES. This can be achieved through various methods such as drop casting [54], spin coating [55], or electropolymerization [42].In the drop casting method, a solution of MIPs is simply dropped onto the surface of the electrode and allowed to dry, forming a thin layer of MIPs. In spin coating, the electrode is rotated at high speed while the MIP solution is dropped onto it, resulting in a uniform thin film. Electropolymerization involves the application of an electric current to initiate the polymerization of monomers directly on the electrode surface. For example, a sensor fabrication involved electrodepositing MIP selectively designed for BPA onto the LSG electrodes (Figure 3) [56]. Electropolymerization was carried out directly on the LSG working electrode surface using pyrrole as the monomer and BPA as the template molecule. The polymerization occurred by cycling the potential between −0.2 V and +0.8 V vs. Ag/AgCl in a solution containing 0.1 M pyrrole and 0.1 mM BPA. The CV scans resulted in the formation of a polypyrrole film imprinted with BPA on the LSG surface. Several parameters were optimized for the electropolymerization including pyrrole concentration, number of cycles, BPA concentration, and incubation time. Increasing pyrrole concentration from 0.05 M to 0.1 M increased the current response and 0.1 M was chosen. The polymerization cycles were increased from 5 to 15, which increased the film thickness until 10 cycles, after which no significant change was observed. Hence, 10 cycles were used. The BPA concentration was varied from 0.05 mM to 0.1 mM, with 0.1 mM giving the highest response. The incubation time after polymerization was optimized to be 2 h. Under these optimized conditions, the imprinting of BPA in the polypyrrole film during electropolymerization resulted in molecular recognition cavities selective for BPA. This enabled sensitive and selective detection of BPA using the LSG-MIES.

In summary, synthesis of MIPs involves forming a pre-polymerization complex between BPA and functional monomers. The pre-polymerization complex is then polymerized using a cross-linker and an initiator. Polymerization techniques include bulk, precipitation, or emulsion polymerization. MIPs are integrated with the electrochemical sensor by drop casting, spin coating, or electropolymerization. Electropolymerization involves applying current to polymerize monomers directly on the electrode surface.

### 2.3. Extraction of BPA Templates

The final step in the fabrication process is the removal of BPA templates from the MIPs. This is typically achieved through washing or extraction with appropriate solvents such as methanol, ethanol, acetic acid, and ethylic acid. The selection of the extraction solution needs to take into account the use of specific monomers. For instance, a study by Rebocho et al. [47] used a supercritical CO_2_-assisted semi-continuous extraction process to remove the BPA templates from the ferrocenylmethyl methacrylate and EGDMA deduced MIP. After MIP synthesis, 5 mL of methanol (co-solvent) was added to the polymer in a high-pressure cell. The cell was heated to 40 °C and CO_2_ was bubbled through to perform a two-step desorption procedure. Firstly, CO_2_ and co-solvent were used to swell the polymer and enhance BPA diffusion. Then, pure CO_2_ was used for washing away residual templates and co-solvent. In report published by Huang et al. [42], after electropolymerization to form the MIPs film using 2-aminothiophenol, the polymer was subjected to a washing procedure in 0.65 M H_2_SO_4_ solution to remove the BPA template entrapped in the polymeric matrix. In report published by Ekomo et al. [30], after synthesizing the MIP using 4-VP, they washed them sequentially with acetonitrile, ethanol-acetic acid solution, and acetone to remove unreacted monomers, the BPA template, and residual monomers/matrix.

The successful removal of BPA templates can be confirmed through various techniques such as UV-visible spectroscopy, HPLC, or mass spectrometry. Rebocho et al. [47] collected the CO_2_ extracts and analyzed them via HPLC to confirm successful removal of BPA from the MIP. They reported 97% removal of BPA after the two-step scCO_2_ extraction process. Ekomo et al. [30] also used HPLC to confirm successful removal of BPA. In addition, they examined the binding sites and cavities in the polymers using nitrogen adsorption/desorption experiments. The surface area and pore volume of MIP were higher than NIP, indicating successful template removal. Huang et al. [42] characterized the electrodes before and after template removal using CV in a solution containing the Fe(CN)_6_^3−/4−^ redox probe. The CV showed a decrease in peak currents after polymerization with the template, indicating the insulating polymer layer. After template removal, the peak currents increased again, suggesting empty binding sites were now available and the redox probe could access the electrode surface. This change in CV response confirmed that the BPA template was successfully removed from the MIPs film after the washing step. Karthika et al. [57] selected FTIR for confirmation. The FTIR spectrum of MIP and NIP confirms the removal of template BPA from MIP cavities. The characteristic peaks of BPA (C-H aromatic stretch at 3050 cm^−1^, C=C aromatic stretch at 1600 cm^−1^, C-O stretch at 1200 cm^−1^) disappeared in MIP spectrum. The pyrrole ring vibration peaks at 1540 cm^−1^ and 1420 cm^−1^ observed in both MIP and NIP. The FTIR spectra showed that the characteristic peaks of BPA were present in the MIP before template removal, but disappeared after the template BPA molecules were removed. This confirmed that the BPA templates were successfully extracted, leaving behind selective imprinted cavities in the MIP film.

The synthesis of MIPs involves forming a pre-polymerization complex between the BPA template and functional monomers via interactions like hydrogen bonding or π–π interactions. The pre-polymerization complex is polymerized using a cross-linker like EGDMA and an initiator like AIBN, using techniques like bulk, precipitation, or emulsion polymerization. MIPs are integrated with the electrochemical sensor using methods like drop casting, spin coating, or electropolymerization directly on the electrode surface. BPA templates are removed from the MIPs by washing with solvents like methanol or acetic acid, which is confirmed using techniques like HPLC, FTIR, or CV.

### 2.4. Types of MIES for BPA Detection

There are various types of MIES designed for the detection of BPA, including potentiometric, amperometric/voltammetric, and EIS sensors. These sensors differ in their mode of operation and the electrical signal they measure.

**Figure 4:**
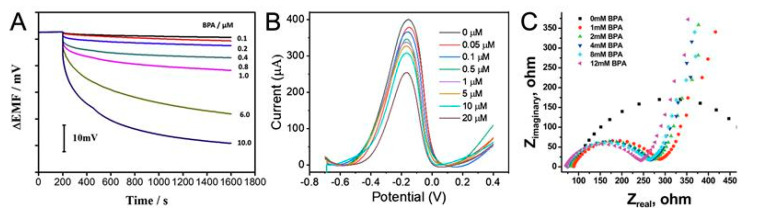
Typical (**A**) potentiometric, (**B**) voltammetric, and (**C**) EIS MIP sensor for BPA detection. Reprinted with permission from Elsevier from Refs. [56,59,60].

Potentiometric MIP sensors operate based on the measurement of the potential difference between two electrodes, a reference electrode, and a working electrode, in the absence of current. The working electrode is typically modified with MIPs that have specific binding sites for BPA. Upon exposure to BPA, the binding of BPA to the MIPs on the working electrode alters the surface potential, which can be measured against the constant potential of the reference electrode. The change in potential is proportional to the concentration of BPA in the sample. For example, a study by Kamel et al. [58] reported a potentiometric MIES for BPA detection. Potentiometric detection was chosen as it provides resistance to interferences from sample color and turbidity, making it promising for real-sample analysis. The key innovation is the use of inexpensive chromatography paper as the electrode substrate rather than expensive materials like glassy carbon. The fabrication process involved patterning hydrophobic wax barriers on chromatography paper using a commercial printer, then coating with conductive carbon ink to create the electrode. The MIP nanobeads were dispersed into the membrane mixture which was drop-cast onto the conductive paper electrode. The paper-based potentiometric sensor exhibited a linear potentiometric response from 0.5–13 μM BPA with a detection limit of 0.15 μM, similarly to the performance of the glassy carbon sensor. Liu et al. [59] developed a novel plasticizer-free potentiometric sensor using a newly synthesized copolymer as the sensing membrane matrix for BPA detection. The copolymer methyl methacrylate-co-2-ethylhexyl acrylate (MMA-2-EHA) was polymerized and used as the membrane matrix. Compared to plasticized PVC, this copolymer membrane exhibited lower cytotoxicity and higher hydrophobicity. The plasticizer-free MIP membrane demonstrated rapid response times within 5 s. It showed good selectivity for BPA over structural analogs like phenol and catechol. Additionally, the MIP sensor had a linear response between 1 μM to 100 μM BPA (Figure 4A), covering the typical concentration range of BPA found in real samples like urine.

Amperometric/voltammetricMIP sensors work on the principle of measuring the current resulting from an electrochemical reaction at a working electrode, which is also modified with MIPs for BPA recognition. The current is directly related to the amount of BPA bound to the MIPs, facilitating the quantification of BPA in the sample. Amperometric/voltammetric MIP sensors are known for their high sensitivity and fast response times. An example of an amperometric MIP sensor for BPA detection is the one developed by Huang et al. [42], which used a BPA-specific MIP film on a AuNPs-modified GCE. The MIP film was synthesized by electropolymerization of 2-aminothiophenol in the presence of BPA as the template molecule. This imprinting process created custom binding sites in the polymer matrix that were complementary to BPA in size, shape, and functional groups. AuNPs were first electrodeposited on the electrode to increase its surface area and current signal. The BPA-MIP film sensor showed excellent performance for quantitative detection of BPA. It displayed good linearity from 8 μM to 60 mM BPA, with a low limit of detection reaching 0.138 μM. Beduk et al. [56] proposed a voltammetric MIP sensor for BPA detection. The sensor showed excellent linearity in the concentration range of 0.05 μM to 20 μM BPA (Figure 4B). The sensor demonstrated a low limit of detection of 8 nM for BPA. The sensor exhibited high selectivity for BPA compared to structural analogs like bisphenol F, bisphenol E, and bisphenol B. The imprinting process creates custom binding sites for only BPA. The relative standard deviation for 10 replicate measurements was calculated to be 2.5% showing good repeatability of the sensor. The sensor retained around 90% of its initial response after 5 reuse cycles. The sensor demonstrated good recovery of 92–108% for BPA spiked in tap water, mineral water, and plastic samples.

EIS is a powerful technique that measures the impedance, or resistance to current flow, of an electrochemical system. EIS-MIES for BPA detection utilize MIP-modified electrodes, and the binding of BPA to the MIPs changes the impedance of the electrode, which can be measured over a range of frequencies. EIS-MIES provides comprehensive information about the electrochemical system, including charge transfer resistance, double layer capacitance, and diffusion characteristics. They offer high sensitivity and can operate in a wide range of conditions. For example, Apodaca et al. [60] electropolymerized a film containing terthiophene and carbazole monomers directly onto an electrode, using BPA as the template. This creates cavities in the polymer film that match the size and shape of BPA molecules. They characterized the electrical properties of the films using EIS (Figure 4C). When the imprinted film is exposed to BPA, the analyte molecules bind to the cavities, changing the interfacial properties of the film. This in turn alters the impedance in a measurable way. The authors obtained a linear calibration curve ranging from 0–12 mM BPA by correlating the changes in impedance to BPA concentration.

In summary, molecularly imprinted electrochemical sensors for BPA detection utilize different transduction modes like potentiometry, amperometry, and EIS along with tailored MIP recognition elements to enable sensitive and selective quantification of BPA. The choice of sensor depends on the specific performance requirements and application.

## 3. Performance Evaluation of Molecularly Imprinted Electrochemical Sensors for BPA Detection

Table 1 shows the performance of recent developed MIES for BPA detection. In addition, the performance of MIES for BPA detection is evaluated based on several key parameters, including sensitivity, selectivity, response time, stability, and reproducibility. These parameters are crucial in determining the effectiveness and reliability of the sensors in real-world applications.

Sensitivity refers to the ability of the sensor to detect small changes in the concentration of BPA, while selectivity is its ability to distinguish BPA from other similar compounds. Both are critical factors in the performance of MIES. The sensitivity of MIES is usually evaluated by measuring the change in the electrical signal (e.g., current, potential, or impedance) per unit change in BPA concentration. For instance, a study by Zhao et al. [51] reported an amperometric MIP sensor for BPA detection with a sensitivity of 0.2871 µA/µM. A sensitivity of 0.4613 µA/µM was achieved by using amino-functionalized GO and MIP for electrochemical sensing of BPA [48].

**Figure 5:**
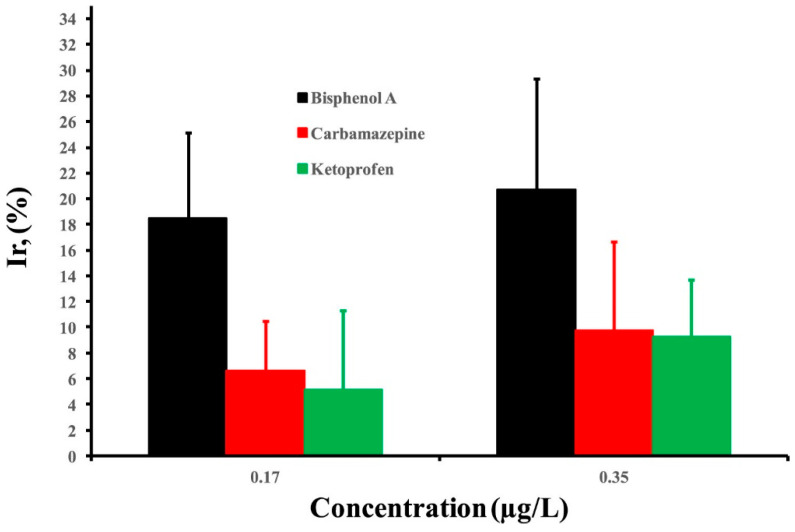
Percentage decrease incurrent intensity (anodic peak) for BPA, carbamazepine, and ketoprofen. Insert: percentage decrease in bar chart. Reprinted with permission from Elsevier from Ref. [30].

Selectivity is typically assessed by comparing the sensor’s response to BPA with its response to other compounds. A highly selective sensor would show a significantly larger response to BPA than to other compounds. For example, the MIP sensor developed by Huang et al. [42] showed a high selectivity for BPA over similar compounds such as phenol, catechol, resorcinol, hydroquinone, and nonylphenol. The results showed that the MIP sensor exhibited the highest current response to BPA compared to the other compounds at the same concentration. The current response to 10 μM BPA was around 250 nA, while the response to 10 μM of the other compounds ranged from around 50 to 100 nA.In another of their studies [61], they tested another MIES with similar compounds. The current response for 1 mM BPA was around 250 μA, while it was less than 80 μA for the same concentration of hydroquinone. Binding experiments showed the imprinted polymer had much higher binding capacity for BPA (35.6 μM/g) compared to structurally similar phenols (1.2–5.4 μM/g). In paper published by Ekomo et al. [30], the proposed BPA sensor gave a significant decrease in peak current for BPA compared to carbamazepine and ketoprofen at the same concentration. At 100 nM concentration, the sensor showed a 60% decrease for BPA, while there was only a 10% decrease for carbamazepine and ketoprofen (Figure 5).

Response time is the time taken by the sensor to reach a steady-state signal after exposure to BPA. A shorter response time is desirable as it allows the rapid detection of BPA, which is particularly important in time-sensitive applications. The response time of MIES can vary depending on the properties of the MIPs and the type of electrochemical detection used. For example, it has been reported that an amperometric sensor can achieve a steady-state current when the scan time was close to 500 s [61]. In most of the scientific literature, authors often emphasize that their sensors possess rapid response times, but they do not always provide specific detection times. This practice can be attributed to several reasons. Firstly, the term “rapid” is relative and can vary greatly depending on the context and the type of sensor being discussed. What is considered “rapid” for one type of sensor might not be the same for another. Therefore, authors often use this term to convey that their sensor’s response time is competitive within its specific field or application. Secondly, the detection time of a sensor can be influenced by a multitude of factors including the nature of the analyte, the concentration, the environmental conditions, and even the specific setup of the experiment. Given these variables, it may not be practical or meaningful to provide a specific detection time that might only be applicable under very controlled conditions. The focus of the research paper might be on the novel method or material used in the sensor design rather than its performance metrics. In such cases, authors might choose to focus their discussion on these aspects and provide a general statement about the response time.

Stability refers to the ability of the sensor to maintain its performance over time, while reproducibility is its ability to give consistent results under the same conditions. Both are important factors in ensuring the reliability of the sensor. The stability of MIES can be affected by various factors such as the stability of the MIPs and the electrode material. For instance, MIPs synthesized from more stable monomers or cross-linkers can enhance the stability of the sensor. In addition, using electrode materials that are resistant to corrosion can also improve stability. Typically, a BPA sensor was constructed by first coating MWCNTs with a silica layer using cetyl trimethyl ammonium bromide (CTAB) and tetraethoxysilane [62]. The silica coated MWCNTs were then functionalized with epoxide groups using glycidoxypropyltrimethoxysilane and vinyl groups using allyl amine. An MIP selective for BPA was synthesized on the surface of the functionalized MWCNTs via copolymerization of functional monomers and a crosslinker in the presence of BPA template molecules. The stability of the sensor was evaluated over a period of 5 weeks. The sensor was stored at 4 °C when not in use. CV scans were performed weekly by immersing the sensor in a 1 μM BPA solution. The oxidation peak currents showed only a gradual decrease over the 5-week period. After 5 weeks, the peak current declined by only 8% compared to the initial response. Cai et al. [63] fabricated a BPA sensor by first covalently attaching amine-functionalized GO, then electrochemically reducing it to graphene. Next, AgNPs were electrodeposited, followed by the co-electrodeposition of polypyrrole and BPA template molecules to form the MIP film. The imprinted sensor showed excellent stability with relative standard deviations of less than 5% for repeatability tests over 10 repeated measurements. The sensor also exhibited good storage stability, retaining 96.2% of its initial current response after being stored dry at 4 °C for 15 days.

Reproducibility is usually assessed by repeating the sensor’s response to a given concentration of BPA multiple times and calculating the relative standard deviation (RSD). A lower RSD indicates higher reproducibility. For example, a study by Dadkhah et al. [48] reported an MIP sensor with an RSD for ten successive determinations of 10 μM BPA less than 4.5%. The fabrication reproducibility was evaluated by comparing the responses of three separately prepared MIP modified electrodes toward 10 μM BPA. The RSD was calculated to be 3.8%, suggesting acceptable reproducibility in the sensor fabrication. In another study [64], an MIP electrochemical aptasensor performed five replicate measurements of a 5 pM BPA solution with a RSD of 4.3%.

When compared with other detection methods for BPA, such as chromatography and mass spectrometry, MIES offer several advantages. They provide direct and real-time detection of BPA without the need for complex sample preparation or expensive instruments. They also offer high sensitivity and selectivity with a wide detection range and fast response times. However, MIES also have their limitations. For instance, they can be affected by environmental factors such as temperature and pH, and their performance can be compromised by fouling or degradation of the MIPs or electrode material. Therefore, continuous efforts are needed to improve the performance and robustness of MIES for BPA detection.

Sensitivity and selectivity are critical performance parameters. High sensitivity allows the detection of low BPA concentrations. Selectivity enables distinguishing BPA from structurally similar compounds. Studies have reported MIES with good sensitivity and selectivity for BPA. Fast response time is desirable for rapid detection. However, response time depends on sensor design and detection method. Authors often qualitatively describe “rapid” response without specifics. Stability and reproducibility affect sensor reliability. Using stable materials enhances stability over time. Reproducibility is assessed by repeatability and fabrication reproducibility. Low relative standard deviations indicate good reproducibility. Compared to other techniques like chromatography and mass spectrometry, MIES offer advantages like simplicity, cost-effectiveness, fast response, and ease of use. However, they can be affected by environmental conditions and material degradation. Overall, while promising, continued research is needed to optimize MIES performance and robustness for practical BPA detection applications.

**Table 1:** Comparison between different MIES for BPA detection.

Sensor	Method	LDR	LOD	Real Sample	Ref.
MIP/AuNPs/MWCNTs	Amperometry	0.11 μM to 8.2 mM	3.6 nM	Honey; Grape juice	[61]
MIP/AuNPs/GCE	Amperometry	8 μM to 60 mM	0.14 μM	Water bottles	[42]
MMIP/SPIONPs/SPCE	Amperometry	25 nM to 0.1 mM	0.16 μM	Saline; Tap water; Mineral water	[65]
MIP/TiO_2_NTs/Ti	Amperometry	4.5 nM to 0.11 μM	2 nM	Water	[66]
MIP/Au/N-MWCNT/GONRs	Amperometry	0.44 μM to 87 μM	8.7 nM	Serum	[67]
MMIP/CTAB/CPE	CV	0.6 μM to 0.1 mM	1 μM	Water bottles; Lake water	[68]
Fe_3_O_4_@TiO_2_/Au/TiMIF	CV	13 nM to 6.6 μM	1.2 nM	Chicken; Pork	[69]
MIP/ABPE	CV	80 nM to 10 μM	60 nM	Plastic	[70]
MIP/GO/GCE	CV	6 nM to 0.1 μM; 0.2 μM to 20 μM	3 nM	Milk; Mineral water	[48]
MIP-AuNPs-MCA-rGO/CILE	CV	4 nM to 15 μM	1 nM	Plastics	[71]
MIP/Au	CV	10 μM to 100 μM	-	-	[72]
MIPMSs/CPE	CV	10 pM to 0.1 μM	2.8 pM	Tap water; Milk	[73]
MIP/graphitic-C_3_N_4_/FTO	CV	5 μM to 0.2 mM	1.3 μM	Bottled water	[74]
MIP(ANI)/GCE	CV	1 fM to 8 fM	0.193 fM	Serum	[75]
MIP/NMWCNT/CPE	CV	0.05 μM to 90 μM	11.8 nM	Plastic bottle leaching	[49]
MIP/PPy@LSG	DPV	80 nM to 5 μM	8 nM	Mineral water; Plastics	[56]
MIM(MIPs)/MWCNTs/GCE	DPV	0.2 μM to 8 μM	8 nM	Tap water; Mineral water	[76]
PEDOT/GQDs/AuNPs/GCE	DPV	1 nM to 50 µM	0.19 nM	Tap water.	[77]
MagMIP-based SPE	DPV	0.1 µM to 10 µM	66 nM	-	[31]
MIPs @ QDs-MWCNTs	DPV	0.025 nM to 50 nM	0.015 nM	Tap water; River water; Drinking water.	[26]
MIP/MWCNT/CPE	DPV	0.1 nM to 0.1 mM	80 pM	Tap water; Baby bottle; Soft drinks; Household filtered water	[45]
MIP/GC	DPV	0.1 nM to 400 μM	0.02 nM	Baby feeding bottle	[62]
MMIP/MGCE	DPV	0.8 μM to 8 μM	0.13 μM	Tea; Milk; Soil; Water	[46]
MIP|ERGO|GCE	DPV	0.5 nM to 750 nM	0.2 nM	Potable water; PC bottled water; Bovine milk;	[57]
rGO-Fe_3_O_4_-ZnOMIP/CPE	DPV	0.008 μM to 15 μM; 15 μM to 95 μM	4 nM	Tap water; Food storage container; Cured vinyl ester resin	[78]
MIP/MWCNTs/CPE	DPV	80 nM to 0.1 mM	22 nM	River water; Tap water	[50]
MIP/rGO/GCE	DPV	5 nM to 0.75 μM	2 nM	Bottled water; Bovine milk	[79]
MIP/PPy/GQDs/GCE	DPV	0.1 μM to 50 μM	40 nM	Sea water; Tap water	[80]
MIP/SPCE	DPV	4.7 nM to 8 nM	3.2 nM	-	[47]
MIP/SPCE	DPV	0.19 nM to 1.8 nM	60 pM	-	[30]
MIP–graphene–Ag/CE	DPV	50 pM to 10 nM	3.2 pM	Plastics	[63]
MIP/Pt/GCE	DPV	7 nM to 0.7 μM	3.2 nM	Serum; Plastics	[81]
MIP/AB/GCE	DPV	5 nM to 0.2 μM; 0.5 μM to 10 μM	2 nM	Bottled water	[82]
MMIP/AuNPs/CNPs/SPCE	DPV	70 nM to 10 μM	8.8 nM	Tap water; Mineral water	[83]
MIP/CNTs/AuNPs/GCE	DPV	10 nM to 10 μM	5 nM	Milk	[84]
β-CD/GO/GCE	DPV	20 nM to 1 μM	8 nM	Drinking water; Lake water	[85]
MIP/MWCNTs/GCE	DPV	0.2 μM to 45 μM	30 nM	Tap water	[86]
MIP/Au-pTH/pABSA/GCE	DPV	80 nM to 0.1 mM	38 nM	River water; Tap water	[87]
MIP/MWCNT/GCE	DPV	0.1 nM to 10 μM	15.7 pM	Plastic bottles; Disposable food boxes;Mobile phone shell	[88]
MIP/GQDs/B-g-C_3_N_4_/GCE	DPV	10 fM to 1 nM	3 fM	Orange juice	[89]
MIP-μPAD	DPV	1 μg/L to 200 μg/L	0.47 μg/L	Water; Plastic bottle water	[90]
CMOF-MIPIL	DPV	5 nM to 5.0 μM	4 nM	Lake water; Plastic bottle; River water; Fresh liquid milk	[91]
GCE/Au/Au@MIP	DPV	0.5 μM to 100 μM	52 nM	Tap water; Milk; Orange juice; Mineral water bottle	[92]
LSG-MIP	DPV	0.01 μM to 10 µM	3.97 nM	Water; Milk; Baby formula; Plastic bottle	[27]
MIP/MWCNTs/CPE	DPV	4 nM to 100 nM; 0.5 μM to 50 μM	4.4 nM	Bottled water	[93]
MIP@CF	DPV	0.5 nM to 8.0 nM; 10 nM to 300 nM	0.36 nM	Milk	[32]
Gr/MIPs/ABPE	DPV	0.321 ng/L to 0.28 ng/L	96.3 pg/L	Plastic pacifier	[94]
PPY/-@p-63/AuNP/GCE	EIS	0.5 fM to 5 pM	0.08 fM	Fresh milk; Milk powder; Tap water	[64]
E-MIP	EIS	1 mM to 12 mM	0.42 mM	-	[60]
MIP/Graphene/ABPE	LSV	8 nM to 1 μM	6 nM	Water bottles; Canned beverages	[95]
MIP/AuNPs/GCE	LSV	15 nM to 55 μM	1.1 nM	Plastic; Milk	[51]
MIP/C-ink/W1C-papes	Potentiometry	0.5 μM to 13 μM	0.15 μM	Plastics	[58]

## 4. Recent Advances

The field of MIES for BPA detection has seen significant advancements in recent years, and there are promising opportunities for further development. These advances and future perspectives can be discussed under three main areas: innovations in materials and fabrication techniques, integration with other technologies, and future challenges and opportunities.

**Figure 6:**
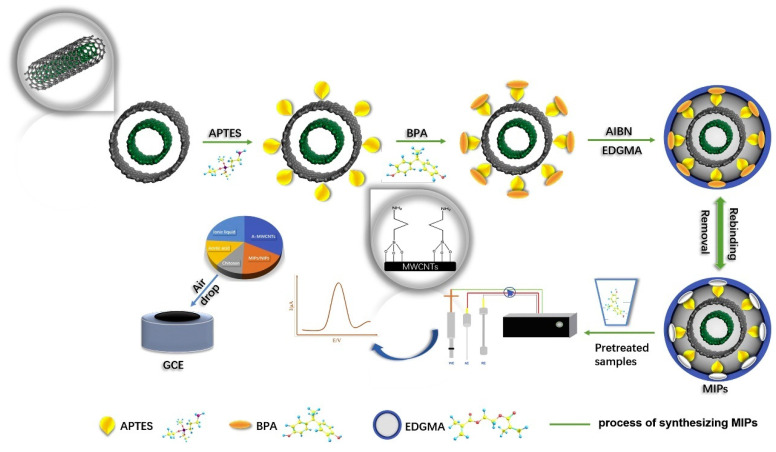
Schematic diagram of the fabrication procedure of doubly oriented functional MWCNTs MIP sensor. Reprinted with permission from Elsevier from Ref. [88].

Recent years have seen exciting innovations in the materials used for MIP synthesis and the techniques used for sensor fabrication. These innovations aim to enhance the performance of MIES in terms of sensitivity, selectivity, response time, stability, and reproducibility. MIES typically utilize polymers that have poor electrical conductivity. Therefore, the addition of nanomaterials with high conductivity can enhance the sensing performance of the sensor. On the other hand, some nanomaterials have unique spatial structures that are beneficial for creating surface morphologies favorable for electrochemical sensing, which can further improve the performance of MIES for detecting BPA. For instance, Zhang et al. [88] proposed a nanocomposite sensor using MIPs based on doubly oriented functional MWCNTs for detection of BPA (Figure 6). The key innovation of this sensor is the use of amino-functionalized MWCNTs as both the carrier and the monomer for the MIPs, rather than adding separate monomers as is traditionally done. This serves two important purposes—the excellent conductivity of the MWCNTs enhances the electrochemical signal, and the amino functional groups increase the number of imprinted sites for selective BPA binding. Computer simulations verified that strong hydrogen bonding interactions can form between the amino groups on the MWCNTs and the hydroxyl groups on BPA. Chitosan was incorporated to further improve the electrochemical response and film-forming capabilities. The resulting nanocomposite sensor showed excellent performance for BPA detection with a wide linear range of 0.1 nM to 10 μM and an ultra-low limit of detection of 15.7 pM. Cai et al. [13] reported the development of a MIES using a nanocomposite material called porous graphene functionalized black phosphorus (PG-BP) for detection of BPA. The authors synthesized PG-BP by combining PG sheets and BP nanoparticles. The PG-BP nanosheets had a wrinkled structure with BP nanoparticles of about 50 nm embedded uniformly throughout the PG matrix. This nanostructure provided a large specific surface area for efficient electron transfer. The PG-BP sensor demonstrated a wide linear detection range from 4.3 × 10^−8^ to 5.5 × 10^−5^ M with a low limit of detection of 7.8 × 10^−9^ M. The practical application of the sensor was demonstrated by accurately determining BPA levels in food package samples and human urine samples without any pretreatment. In work reported by Zhao et al. [51], AuNPs were used for MIES fabrication. The paper mentions that the AuNPs could significantly increase the conductivity of the MIES. Higher conductivity facilitate better electrochemical performance of the sensor. To provide a suitable surface for self-assembly of the MIES, the 4-aminothiophenol monomer was self-assembled onto the AuNPs modified electrode via Au-S bonds. To promote electron transfer for the electrochemical reaction, the AuNPs can accelerate the electron transfer kinetics of the redox probe at the electrode interface, enhancing the electrochemical signal.

The integration of MIES with other technologies such as microfluidics is another promising area of development. This integration can enhance the functionality and applicability of the sensors. Nanotechnology can be used to improve the sensitivity and selectivity of MIES through the use of nanostructured materials. For example, a study by Jemmeli et al. [96] reported a paper-based electrochemical sensor for BPA detection. The key purpose of using paper-based microfluidics is to create a portable, disposable, and eco-friendly sensor that can provide rapid decentralized testing of BPA at the point of need. The paper support eliminates complex pumps and tubing, while allowing onboard storage of reagents within the porous network. The wax printing facilitates simple fabrication of the microfluidic electrochemical cell. The sensor shows excellent performance for BPA sensing with a low detection limit of 0.03 μM and two wide linear ranges of 0.1–0.9 μM and 1–50 μM. The analysis of river water and drinking water samples demonstrate its ability to accurately detect BPA in real water matrices.

## 5. Future Perspectives and Conclusions

In conclusion, this paper has reviewed the emerging technology of MIES for BPA detection. MIES show great promise as a selective, sensitive, cost-effective approach for on-site BPA analysis. However, there remain challenges to be addressed before MIES can realize their full potential.

One major challenge is improving selectivity in complex real-world samples containing interfering compounds. Techniques like liquid–liquid extraction [97,98] or solid-phase extraction [99,100] are used to isolate BPA from complex sample matrices. This removes potential interferents and concentrates BPA for improved detection. BPA is sometimes chemically derivatized before analysis to improve its detection properties. Common derivatization techniques include acetylation [101], methylation [102], or silylation [103]. Enrichment techniques like solid-phase extraction or immunoaffinity extraction can selectively concentrate and isolate BPA from samples prior to analysis. This improves detection limits. Further research on novel monomers, functionalization strategies, and imprinting methods is needed to enhance specificity towards BPA over structurally similar compounds.

Another key challenge is reproducibility and standardization of MIP synthesis and sensor fabrication. Variations in imprinting conditions and fabrication procedures can lead to inconsistencies in sensor performance. Developing robust protocols for MIP preparation and strict quality control during sensor manufacturing is critical for reliable performance. Techniques like covalent grafting of MIPs onto sensor surfaces may improve uniformity and stability.

Looking ahead, emerging materials like graphene composites and advanced imprinting techniques provide new opportunities to overcome current limitations. With further research to address selectivity and reproducibility issues, MIES have immense potential for decentralized, on-site BPA monitoring across a range of applications from food safety to environmental analysis. By enabling rapid, cost-effective BPA detection, widespread adoption of MIES can significantly reduce human and environmental exposure to this endocrine-disrupting chemical. In summary, integrating molecular imprinting with electrochemical transduction is a highly promising approach for selective BPA detection, but requires continued research to fully realize its potential. Overcoming current challenges will pave the way for field-based MIES that can deliver significant societal benefits.

## Data Availability

Data sharing not applicable.
